# Development of a luciferase-based reporter system to monitor *Bifidobacterium breve *UCC2003 persistence in mice

**DOI:** 10.1186/1471-2180-8-161

**Published:** 2008-09-24

**Authors:** Michelle Cronin, Roy D Sleator, Colin Hill, Gerald F Fitzgerald, Douwe van Sinderen

**Affiliations:** 1 Alimentary Pharmabiotic Centre, University College Cork, Western Road, Cork, Ireland; 2 Department of Microbiology, University College Cork, Western Road, Cork, Ireland

## Abstract

**Background:**

Probiotics such as bifidobacteria have been shown to maintain a healthy intestinal microbial balance and help protect against infections. However, despite these benefits, bifidobacteria still remain poorly understood at the biochemical, physiological and especially the genetic level. Herein we describe, for the first time, the development of a non-invasive luciferase-based reporter system for real-time tracking of *Bifidobacterium *species *in vivo*.

**Results:**

The reporter vector pLuxMC1 is based on the recently described theta-type plasmid pBC1 from *B. catenatulatum *[1] and the *luxABCDE *operon from pPL2lux [2]. Derivatives of pLuxMC1, harbouring a bifidobacterial promoter (pLuxMC2) as well as a synthetically derived promoter (pLuxMC3) [3] placed upstream of *luxABCDE*, were constructed and found to stably replicate in *B. breve *UCC2003. The subsequent analysis of these strains allowed us to assess the functionality of pLuxMC1 both *in vitro *and *in vivo*.

**Conclusion:**

Our results demonstrate the potential of pLuxMC1 as a real-time, non-invasive reporter system for *Bifidobacterium*. It has also allowed us, for the first time, to track the colonisation potential and persistence of this probiotic species in real time. An interesting and significant outcome of the study is the identification of the caecum as a niche environment for *B. breve *UCC2003 within the mouse gastrointestinal tract (GI) tract.

## Background

The human small and large bowel accommodates bacteria belonging to more than 400 known species, many of which play a mutualistic role in the digestion of dietary nutrients [4]. The concept of probiotic bacteria has evolved from a live active culture which improves the balance of the gut microbiota composition, to one that incorporates specific beneficial effects, including maturation of the mucosal adaptive immune system [5]. Indeed consumption of probiotics such as bifidobacteria has been shown to maintain a healthy intestinal microbial balance and help protect against intestinal infections [6-8]. A significant goal of current research is to understand the complex nature of the probiotic interaction with the innate immune system. This ability to direct and modulate the immune system has significant implications in the areas of atopic disease, cancer and pathogenesis [9]. However, despite these potential beneficial properties and a growing industrial and consumer acceptance, bifidobacteria still remain poorly understood at the biochemical, physiological and especially the genetic level [10,11]. Current efforts in the field of bifidobacterial genetics are focusing on developing vectors for the genetic manipulation of the species *in vitro *[12-14].

In order to confirm a strain-specific probiotic action it is necessary to associate a given health-promoting function with expression of a particular gene product(s). Gene reporter fusions have been used for decades to identify and monitor gene expression [15-18]. Traditional reporter systems, based on enzymatic assays, are often limited to *in vitro *retrospective analysis, typically involving cell disruption and addition of an enzymatic substrate followed by an absorbance measurement. To date, the promoterless *gusA *gene is the only successful reporter system utilized for *Bifidobacterium *[13,18-20] and although valuable for *in vitro *analysis of gene expression, it is limited by the fact that it is semiquantative and cannot be applied in an *in vivo *model.

A novel reporter system based on quantified light emission (bioluminescence) as a result of *lux *gene expression has achieved considerable attention in recent times [21]. Light generation in naturally occurring bioluminescent bacteria is encoded by five essential genes organized in an operon such as *luxCDABE*. Blue-green light is emitted from these bacteria with a peak at 490 nm as a result of a heterodimeric luciferase, an enzyme which catalyses the oxidation of reduced flavin mononucleotide (FMNH_2_) and a long-chain fatty aldehyde (synthesized by a fatty acid reductase complex encoded by *luxCDE*). Although a number of additional *lux *genes have been identified, only *luxA-E *are essential for the biosynthesis of light [22]. To date the *lux *operon has been successfully expressed in a variety of Gram-negative bacteria, conferring a bioluminescent phenotype [23]. Since all identified species of naturally occurring marine and terrestrial bioluminescent bacteria are Gram-negative, the generation of Gram-positive bioluminescent bacteria has been limited. However, the introduction of Gram-positive ribosome binding sites and shuffling of the gene order to *luxABCDE *[24] has facilitated the successful application of the luciferase system in a limited number of Gram-positive bacteria [22,25,26].

A recent study by Guglielmetti [27] reported the use of insect luciferase in *B. longum *biovar *longum *as a biosensor to analyze the metabolic state of cells. Herein we describe for the first time the development of a Lux (bacterial luciferase) based reporter system for the species *Bifidobacterium*. The reporter vector pLuxMC1 is based on the recently described theta based plasmid pBC1 from *B. catenatulatum *[1] and the *luxABCDE *operon from pPL2lux [2]. Derivatives of pLuxMC1, harbouring a bifidobacterial promoter (pLuxMC2) as well as a synthetically derived promoter (pLuxMC3) [3] placed upstream of *luxABCDE*, were constructed and found to stably replicate in *B. breve *UCC2003, a strain originally isolated from nursling stool.

The subsequent analysis of these strains allowed us to assess the functionality of pLuxMC1 both *in vitro *and *in vivo*. Our results demonstrate the potential of pLuxMC1 as a real-time, non-invasive reporter system for *Bifidobacterium*. It has also allowed us, for the first time, to track the colonisation potential and persistence of this probiotic species in real time. An interesting and significant outcome of the study is the identification of the caecum as a niche environment for *B. breve *UCC2003.

## Results

### Construction of the luciferase based reporter system pLuxMC1

**Figure 1:**
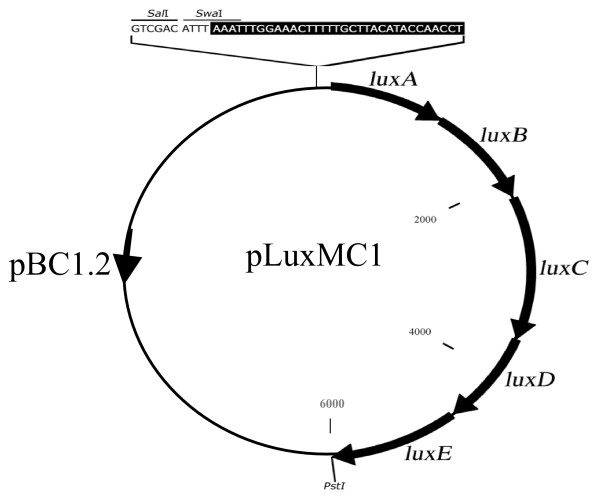
**Plasmid map of pLuxMC**. pBC1.2 is bifidobacterial shuttle vector containing a chloramphenicol-resistance cassette [14]. The *luxABCDE *operon with a blunt SwaI restriction site overlapping the *luxA *start codon was derived from pPL2*lux *[2].

The pLuxMC1 vector (Fig. 1) is a derivative of the shuttle vector pBC1.2 [14] that contains a putative theta-type replication origin and a chloramphenicol (Cm) resistance marker. In addition, pLuxMC1 contains *lux*ABCDE, a synthetic operon that contains optimized Gram-positive translational initiation sequences upstream of *luxA, luxC and luxE *[24]. The *lux*ABCDE operon is divergently orientated with respect to flanking genes on pLuxMC1 to minimize any potential read-through from upstream promoters. Importantly, the use of the SalI and SwaI restriction sites for cloning of PCR-amplified promoters into pLuxMC1 allowed the creation of transcriptional fusions to the *lux*ABCDE operon. Furthermore, since the ribosomal binding site and start codon of *luxA *are absent in pLuxMC1, such fusions are also required to include appropriate translational start signals, and thus allow direct identification of correct expression signal insertions by their bioluminescent phenotype in *E. coli*.

### Functionality and stability of pLuxMC1 derivatives

To demonstrate the functionality of the pLuxMC1 luciferase reporter system in *B. breve *UCC2003, the expression profiles of two promoters and associated translational signals were assessed. The first of these (P_*rep*_) is the promoter driving the expression of the *repC *gene from the cryptic *B. catenulatum *plasmid pBC1. The second is a constitutive optimised promoter (P_*help*_), from *Listeria monocytogenes*, where it has been shown to elicit constitutively high activity [3]. Vectors pLuxMC2 and pLuxMC3 containing each of these promoters respectively, were constructed in *E. coli *and transformed into *B. breve *UCC2003 as described in Materials and Methods.

The stability of all constructs in the absence of antibiotic selection pressure was evaluated. Cultures of *B. breve *UCC2003 containing pLuxMC1, pLuxMC2 or pLuxMC3, were grown for 100 generations in MRS medium without antibiotic selection, after which dilutions were plated every 20 generations to assess the presence of the plasmids in the resulting colonies by scoring for Cm resistance encoded by the pBC1.2 backbone. All 100 colonies tested for each of the three strains at each of the time points were Cm-resistant (data not shown), indicating that the plasmids were stably maintained without antibiotic selection pressure for at least 100 generations under the conditions tested. This high level of stability allowed use of the luciferase reporter system in animal experiments in which antibiotic selection pressure could not be maintained.

**Figure 2:**
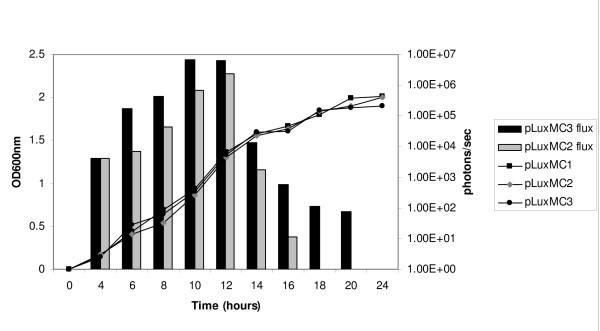
**Growth and expression profiles for *B. breve *UCC2003 pLuxMC derivatives**. Growth and expression of UCC2003 pLuxMC1, pLuxMC2 and pLuxMC3 during growth at 37°C in MRS medium supplemented with cysteine-HCL and chloramphenicol. The line graphs indicate the average growth of triplicate cultures and the grey and black bars indicate the average luciferase expression profiles for pLuxMC2 and pLuxMC3 respectively. BLU is the bioluminescence counts measured as photons per second; OD600 nm is the optical density at 600 nm. The data are representative of the data from three independent experiments.

Overnight cultures of the pLuxMC-containing *B. breve *strains were diluted 1:50 in MRS medium supplemented with cysteine HCl. Growth and bioluminescence were then monitored over time (Fig. 2). No difference in growth rates or final optical density were detected between *B. breve *UCC2003 harbouring either pLuxMC2, pLuxMC3 or pLuxMC1, indicating that luciferase expression at the levels reached during the experiments described here does not influence the growth rate of *B. breve *UCC2003. No bioluminescence was detected at any time for the control strain *B. breve *UCC2003 containing pLuxMC1, demonstrating the absence of any background signal (data not shown). For the *B. breve *strain harbouring pLuxMC3, bioluminescence was detected throughout all growth stages, indicating constitutive expression of the *lux *operon (Fig. 2). In contrast, for pLuxMC2 bioluminescence declined during the late logarithmic phase of growth and further decreased in the stationary phase, until the signal eventually became undetectable. This significant difference in bioluminescence profiles observed for pLuxMC2 and pLuxMC3 during stationary phase suggests that differential expression can be monitored in *B. breve *UCC2003 using the luciferase reporter system.

### Expression in a murine model

**Figure 3:**
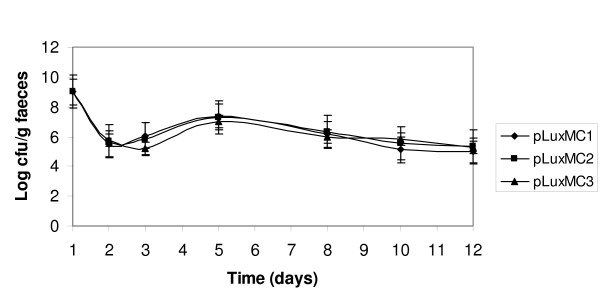
***B. breve *UCC2003 pLuxMC1-3 recovered from murine stool**. Recovery of *B. breve *UCC2003 pLuxMC derivatives from murine stool samples over a 12 day trial period. The data are representative of the average colony forming units per gram of faeces from three faecal samples per group per time point (days).

**Figure 4:**
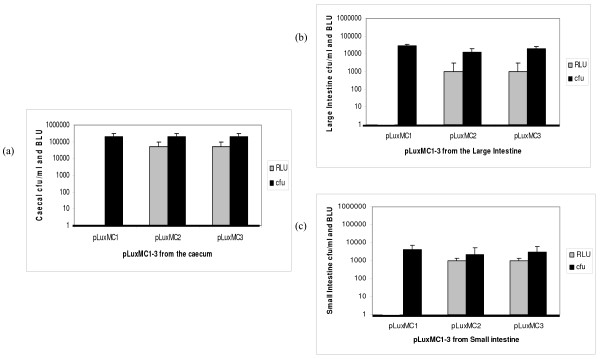
**Comparison of pLuxMC1-3 colony forming units and bioluminescence from murine GIT samples nine days post-inoculation**. Comparison of *B. breve *UCC2003 pLuxMC derivatives recovered from (a) the caecum; (b) the large intestine and (c) the small intestine, each set of data is representative of the average values from three mice per group. The BLU (bioluminescence counts in photons per second) represented in grey refers to the bioluminescence recorded from the intact tissue sections upon excision 9 days post-inoculation and immediately prior to homogenization. The black bar is the average CFU (colony forming units) per ml of homogenized tissue sample.

The suitability of the pLuxMC1 reporter system to monitor gene expression *in vivo *was investigated in the gastrointestinal tract of mice. Three groups of three mice were orally inoculated with *B. breve *UCC2003 containing either pLuxMC1, pLuxMC2 or pLuxMC3. Whole body image analysis was performed 5 days following the final inoculation. No distinct luminescent signal was observed for any of the groups which may be explained by the findings of Rice *et al*, [28] who reported that cell numbers in excess of 10^6 ^are required to detect signals at 2 cm depth in tissue. Shedding of *B. breve *UCC2003 containing the pLuxMC derivatives in the faeces was detected throughout the twelve-day trial period. The administered bacterial populations increased in number in the faecal samples up to day 5, reaching a plateau at approximately 10^7 ^cfu g^-1 ^stool, declining to around 10^5 ^cfu g^-1 ^stool at day 12 post inoculation (Fig. 3). Nine days following initial inoculation the mice were sacrificed by cervical dislocation, and the levels of bioluminescence in the intact GI tracts were determined and correlated with the numbers of bacterial cells recovered on selective medium (Fig. 4). Notably, the numbers of CFU recovered from the mice inoculated with all three strains were very similar, suggesting that luciferase expression does not influence the persistence of *B. breve *UCC2003. The results also confirm that the bioluminescence data is quantitative, correlating with the number of bacteria present. However, viable counts were found to have higher limits of detection than luminometry. No bioluminescence was detected in the GI tracts of the three mice in the control group. In contrast, the caecum obtained from the two groups of mice fed with either *B. breve *UCC2003 pLuxMC2 or UCC2003 pLuxMC3 displayed relatively high levels of bioluminescence. The detection limits of the IVIS100 imaging system (Xenogen) were determined to be approximately 10^3 ^bacteria within a given focus, which correlates well with previous studies [29]. Interestingly, in contrast to the *in vitro *expression profile, both vectors were detected to an equal extent in the gastrointestinal tract after 12 days. The data clearly demonstrate the usefulness of the pLuxMC reporter system to monitor bifidobacterial gene expression in the murine GI tract.

### Dynamics of colonization and persistence of *B. breve *UCC2003

**Figure 5:**
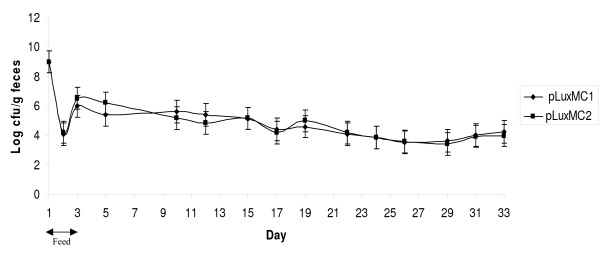
**Persistence of *B. breve *UCC2003 pLuxMC derivatives in the murine GIT**. The log of colony forming units of *B. breve *UCC2003 containing either pLuxMC1 (●) or pLuxMC2 (■) per gram of faeces over 33 day trial period. The data is representative of duplicate plating from the available feacal samples at each given time point. On day one to three the mice were orally gavaged with 10^9 ^*B. breve *UCC2003 containing either pLuxMC1 (control) or pLuxMC2 (test).

Colonization and clearance of *B. breve *UCC2003 harbouring pLuxMC1 (hereafter referred to as control) or pLuxMC2 (test) was monitored over a thirty-three day period by following bioluminescence and viable counts recovered from both the GI tract and faecal samples. Two groups of mice (n = 5) were orally inoculated with either the control or test strain. Shedding of *B. breve *UCC2003 control and test strains in the faeces was detected throughout the trial with no statistically significant differences observed between the two groups. The inoculated bacterial population increased in number (Fig. 5), reaching a maximum of ~10^7 ^cfu g^-1 ^faeces at day 5, from day 10–19 the level of UCC2003 shed plateaus at ~10^5 ^cfu g^-1 ^faeces. Following day 19 there is a steady decline to ~10^4 ^cfu g^-1 ^until days 31 and 33 when a marginal increase in faecal shedding was observed.

On day 5, the GI tract recovered from a single test animal showed detectable bioluminescence originating from both the lower small intestine and the caecum, with little signal detectable in the colon. Viable counts of both stool and tissue samples taken on day 5 from both test and control fed mice revealed that *B. breve *UCC2003 was present within the colonic mucosa, suggesting that at this time point microcolonies, consisting of at less than 10^3 ^bacteria had formed. Within 2 days of inoculation, the majority of the bolus of bacteria had travelled through the gut, with some of the inoculum remaining in the lower small intestine; bioluminescence on day 5 from this portion of the GI tract corresponded with the presence of a large food bolus. After the initial bolus had passed no detectable luminescence was observed from the small intestine and the level of bacteria detected by viable counts fell to ~10^3 ^cfu g^-1^.

**Figure 6:**
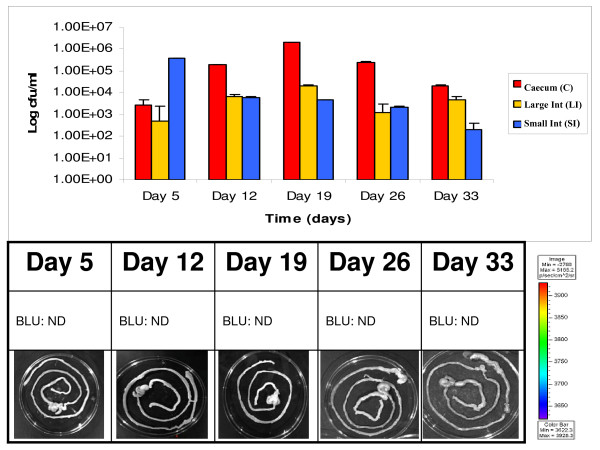
**Correlation between bioluminescence and colony forming units (pLuxMC1)**. Illustrates the log colony forming units per ml from groups of mice feed pLuxMC1 for three days followed by thirty days of persistence study. The recovered gastrointestinal tracts (GIT) at each of the five time points were divided into the major physical sections and the average cfu/ml from the homognised tissue samples are represented in red (■) for the caecum, yellow (■) for the large intestine and the small intestine in blue (■). The image panel in both (a) and (b) represents an intact excised GIT at each time point. These images were obtained using an IVIS 100 system with 5 min of exposure and a binning value of 8. The color bar indicates the bioluminescence signal intensity (in photons s^-1 ^cm ^-2 ^sr^-1^). ND, not detectable (bioluminescence counts less than 1 × 10^4 ^photons s^-1^). BLU, bioluminescence counts (photons s^-1 ^cm ^-2 ^sr^-1^); BLU C, recovered from caecum; BLU LI, from the large intestine and BLU SI from the small intestine.

**Figure 7:**
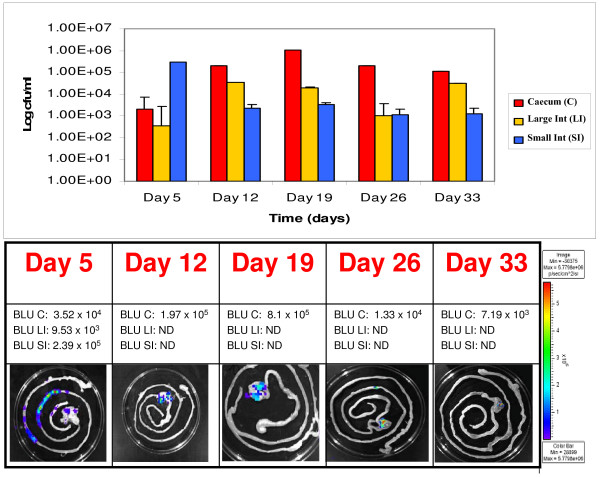
**Correlation between bioluminescence and colony forming units (pLuxMC2)**. Illustrates the log colony forming units per ml from groups of mice feed pLuxMC2 for three days followed by thirty days of persistence study. The recovered gastrointestinal tracts (GIT) at each of the five time points were divided into the major physical sections and the average cfu/ml from the homognised tissue samples are represented in red (■) for the caecum, yellow (■) for the large intestine and the small intestine in blue (■). The image panel in both (a) and (b) represents an intact excised GIT at each time point. These images were obtained using an IVIS 100 system with 5 min of exposure and a binning value of 8. The color bar indicates the bioluminescence signal intensity (in photons s^-1 ^cm ^-2 ^sr^-1^). ND, not detectable (bioluminescence counts less than 1 × 10^4 ^photons s^-1^). BLU, bioluminescence counts (photons s^-1 ^cm ^-2 ^sr^-1^); BLU C, recovered from caecum; BLU LI, from the large intestine and BLU SI from the small intestine.

While *B. breve *UCC2003 numbers remained detectable by viable colony counts, verified by colony PCR, for both the small intestine and colonic samples at day 12, 19, 26 and day 33 the counts remained close to 10^3 ^cfu g^-1 ^tissue which is below the limits of bioluminescence detection of the Xenogen IVIS100 system for intact organs. Interestingly, the caecum remained colonised throughout the trial (Fig. 6, Fig. 7). Once again bioluminescence data strongly correlated with the number of bacteria present in homogenized tissue samples. Colonisation of the caecum was evident from day 5 and by day 12 the caecum was the only portion of the GI tract where bioluminescence could be detected corresponding to a log increase in the number of CFU. Colonisation of the caecum peaked at day 19 and by day 33 the caecum was the only portion of the GI tract containing significant numbers of the inoculated bacteria (10^5 ^cfu g^-1^). The reduced levels of *B. breve *UCC2003 observed in the caecum at day 33 correlated with a marginal increase in shedding observed on day 31 and 33 possibly reflecting the clearance of the administered *B. breve *UCC2003 from the murine GI tract (Fig. 5).

### Contribution of the native bifidobacterial population

Previous studies have reported native bifidobacterial populations in the murine GI tract of 10^3 ^cfu g^-1 ^for the small intestine and 10^5 ^cfu g^-1 ^for the colon including the caecum [4]. To establish if this trend was evident throughout the persistence study, aliquots of homogenised tissue samples were plated on RCA containing mupirocin, which has been previously reported as a selective agent for *Bifidobacterium *[30]. Total bifidobacterial counts from the caecum followed a similar trend to that of the inoculated bacteria levels, beginning at 10^5 ^on day 5, rising to 10^8 ^by day 19 before reducing to 10^5 ^at day 33. The bifidobacterial population in the colon remained relatively constant at ~10^5 ^while the small intestine showed an initial total population of 10^6 ^on day 5 followed by a plateau at ~10^4 ^throughout the remainder of the trial. Random colonies displaying bifidobacterial morphology which were not chloramphenicol resistant were further analysed, chromosomal DNA was extracted and a PCR targeting the 16S intergenic spacer region [32] allowed us to identify five species of bifidobacteria native to these murine intestines with blast homology to *B. longum, B. adolescentis *(small intestine), *B. animalis *and *B. animalis *subsp *lactis*.

## Discussion

In this study we describe the construction of a luciferase-based reporter system, pLuxMC1, and the successful application of this system to track *B. breve *UCC2003 growth *in vitro *(laboratory medium) and *in vivo *(murine model). The vector pLuxMC1, similar to pPL2lux [2], allows for the construction of exact translational/transcriptional fusions of promoters to the luciferase-encoding genes. As consensus promoters in the genus *Bifidobacterium *are not well defined, we chose to evaluate a native bifidobacterial expression signal (P_*rep*_), which is located upstream of *repC *on the cryptic plasmid pBC1 and which was previously shown to be essential for pBC1 replication [14]. We also examined P_*help *_[3], a promoter developed for constitutive gene expression in *L. monocytogenes *and a wide range of Gram-positive and Gram-negative species [31]. Both of the luciferase-expressing *B. breve *strains displayed growth characteristics similar to those of control strains and no background bioluminescence was observed during any of the experiments. This highlights the major advantage of luciferase-based reporter systems when compared to the glucuronidase-based reporter systems currently available for Bifidobacteria [13,18-20].

One potential caveat to the use of the luciferase-based system for *B. breve *is that the oxygen requirements for the effective functioning of LuxABCDE have to be fulfilled by an anaerobic bacterium in a supposedly anaerobic environment [23]. The oxygen dependence of bacterial luciferase has previously been critically studied in light of certain *in vivo *imaging applications and we concur with Hardy *et al. *[25] that the presence of a luminescent signal at a particular site indicates that sufficient oxygen is present for the reaction to occur, but that bacterial counts should in the first instance be determined to confirm that the level of luminescence is correlated to a certain number of cells under a given set of experimental conditions. The determination of a direct correlation was not possible through whole-body imaging of *B. breve *UCC2003-inoculated mice and indeed this analysis may not be possible using the current system as, regardless of the light source, transmission depends on the type of tissue and the depth of the light source in the animal [29]. Future efforts to obtain such images may involve imaging the mice at a later stage of colonization, increasing *lux *expression levels or increasing the cell densities through the exploitation of bifidobacterial-specific growth promoting factors such as non-digestible oligosaccharides [33].

The ability of *B. breve *UCC2003 to express a *Lux*^+ ^phenotype *in vivo *was initially investigated over a period of twelve days using bioluminescence as well as viable count measurements. Analysis of the two pLuxMC-containing strains revealed that the *in vitro *expression profile was not reflected *in vivo*. The P_*help *_promoter of pLuxMC3 gives significantly higher bioluminescence expression to P_*rep *_of pLuxMC2 (Fig 2) during stationary-phase growth under laboratory conditions; however, under *in vivo *conditions the difference in expression is no longer apparent indicating that in the murine model the native bifidobacterial promoter P_*rep *_is as suitable to drive luciferase expression as the constitutive promoter P_*help*_.

The luminescence of microbial cells is strongly dependent on metabolic activity, making the *Lux*^+ ^phenotype an indicator of metabolic integrity [34]. During *in vitro *growth of many bacterial species harbouring the *lux *operon, bioluminescence declines when cells enter stationary phase [26]. This pattern of light production was observed *in vitro *for *B. breve *UCC2003 containing pLuxMC2 and this may be caused by a decrease in metabolic activity. The tight correlation between luminescence and viable counts obtained from both stool and organ homogenates during colonisation of mice with *B. breve *UCC2003 suggests that, similar to what has previously been reported for *Citrobacter rodentium *[29], colonizing bacteria are metabolically similar to those seen during the exponential phase of *in vitro *growth and that intracellular cofactors are not limiting.

The bioluminescent *B. breve *UCC2003::pLuxMC2 was subsequently exploited to investigate *in situ *and in real time, the dynamics of colonization of orally inoculated mice over a 33-day period. The short half-life (several seconds) of the luciferase enzyme [35], coupled with a very low level of background luminescence in mammalian tissue makes bioluminescence an excellent reporter system for analysing colonization of a host intestine by bacteria as observations occur in real time and do not reflect accumulated signal.

The most striking finding of this study is that primary colonization of the mouse by *B. breve *UCC2003 takes place within the caecum. Although bifidobacterial species have previously been isolated at 10^5 ^CFU/g from pig caecal contents [30], 10^3 ^CFU/g from rabbit [36], 10^6 ^CFU/ml from human [37], and 10^9 ^CFU/g from hen [36], this is the first time that the caecum has been implicated as the predominant colonization site for these bacteria. Interestingly, pathogens such as EHEC O157:H7 [38] and *C. rodentium *[29] also exhibit murine caecal colonization. Histologically the organisation of the caecum is similar to Peyer's patches (PPs) with domed villi containing M cells and dendritic cells. A variety of pathogens exhibit a tropism for PP. Following adhesion, enteroinvasive *Yersinia*, *Shigella*, and *Salmonella *sp. enter PP and are able to multiply within this tissue [39]. In *S. typhimurium *targeting of the pathogen to PP is mediated by surface components including the long polar fimbriae which when inactivated impairs the colonization of murine PP [40]. The first stage of colonization within the caecal patch of the mouse intestine by *C. rodentium *is also proposed to be mediated by the synergistic action of fimbriael operons [41].

The caecum may be the site which allows certain pathogens to adapt to the intestinal environment, and where genes required for efficient colonization of the colon are activated [42]. The caecum may also act as a reservoir, shedding bacteria into the colon. It is interesting to note that for *C. rodentium *the caecal patch is also the first site to be cleared of infection and that clearance of the colon follows shortly afterwards [29]. Evidence exists that the best protection against mucosal attachment and invasion by such pathogens is by keeping intestinal microbiota in a state that affords colonisation resistance against pathogens by modulation of the microbiota inducing luminal or systemic effects which are beneficial to the host's health [43]. It is conceivable that *B. breve *UCC2003 may act through competitive exclusion in the caecum, at least in the murine model, and that their presence may prevent pathogenic bacteria from becoming established.

## Conclusion

The application of pLuxMC1 derivatives has significant potential to allow further study of the interaction of bifidobacteria with a mammalian host. The system has the distinct advantage of allowing direct comparison of different promoter activities in intact animal tissue. This reporter system is a valuable addition to the arsenal of genetic tools available for bifidobacteria as it can be employed for *in situ *real-time investigation of promoter activities both *in vitro *and *in vivo*. Currently, the luciferase reporter system is being exploited in our laboratory to explore expression profiles for *B. breve *UCC2003 chromosomal genes to further our understanding of transcription signals in this strain (unpublished data). The wealth of information regarding gene expression that will become available from this type of approach is expected to contribute significantly to understand the molecular mechanisms underlying *B. breve *UCC2003 behaviour *in situ*.

## Methods

### Bacterial strains, plasmids and culture conditions

Bacterial strains, plasmids and primers used in this study are listed in Table 1. *E. coli *strain TOP10 (Invitrogen, Paisley, United Kingdom) was used as a cloning host for the construction of pLuxMC1 and its derivatives (see below) and was grown aerobically at 37°C in LB medium [44]. *B. breve *UCC2003 was routinely grown at 37°C in reinforced clostridial medium (Oxoid, Basingstoke, United Kingdom). However, for bioluminescence assays MRS medium (Oxoid), supplemented with 0.05% (w/v) cysteine-HCl was used. Anaerobic conditions were maintained using an anaerobic chamber [Mac500, Don Whitley Scientific, West Yorkshire, UK (atmosphere 10% H_2_, 10% CO_2_, 80% N_2_)]. Where appropriate, antibiotics were added to the growth media at the following concentrations: for *E. coli*, ampicillin at 100 μg ml^-1 ^or chloramphenicol at 20 μg ml^-1^; for *B. breve*, chloramphenicol at 4 μg ml^-1^. To facilitate specific recovery of bifidobacteria from intestinal samples, 50 mg of mupirocin (Oxoid)/liter was added to reinforced clostridial agar (Oxoid) from antimicrobial susceptibility test discs, as previously described [30].

**Table 1:** Bacterial strains, plasmids and primers used in this study.

**Bacterial strains**	**Relevant properties**	**Source/Reference**
*Escherichia coli *TOP10	F-*mcr*A φ80*lac*Z_M15 *lac*X74*rec*A1*ara*D *gal*U *rps*L *end*A1 *nup*G	Invitrogen, UK.
*Bifidobacterium breve *UCC2003	Electroporation host	UCC Culture Collection.

**Plasmids**	**Relevant properties**	**Source/Reference**

pPl2lux	Allows for translational fusions to *lux*ABCDE operon	[2]
pUC19	2.686 kb vector based on pMB1, Amp^r^	Fermentas
pUC19-*lux*	pUC19 containing the 5.6 kb modified *lux*ABCDE operon as SalI – PstI fragment.	[2]
pBC1.2	Bifidobacterial shuttle vector based on pBC1, Cm^r^	[14]
pLuxMC1	pBC1.2 containing the modified *lux*ABCDE operon	This study
pLuxMC2	pBC1.2 containing the *lux*ABCDE operon plus promoter of *repC *from pBC1	This study
pLuxMC3	pBC1.2 plus *lux*ABCDE operon with P_*help *_[3].	This study

**Primers**	**Sequence**	**Source/Reference**

IM111	Aaaaggacgatttcggttgg	[2]
IM112	Ccaatgccccagaaatttcc	[2]
P_*rep *_F	Ccatccaactcgaggcacaagccgcgcgagcggtc	This study
P_*rep *_R	Catgggcactagtgtacgtc	This study

Amp^r^, ampicillin resistance; Cm^r ^chloramphenicol resistance. Underlining indicates restriction sites used in subsequent cloning steps

### DNA techniques

Plasmid DNA was isolated from *E. coli *using a QIAprep Spin Miniprep kit according to the manufacturer's instructions (QIAGEN, Crawley, United Kingdom). Genomic DNA isolation and transformation of *B. breve *UCC2003 were performed as described previously [45]. Standard procedures were used for DNA manipulation in *E. coli *[46]. Restriction endonucleases (Roche Diagnostics, Mannheim, Germany), T4 DNA ligase (Roche), and 2× PCR mixture (Promega, Madison, WI) were used as recommended by the manufacturers. Primers were purchased from MWG (Ebersberg, Germany) and are listed in Table 1. PCR products that needed to be cloned were obtained with KOD hot-start high-fidelity DNA polymerase (Merck, Nottingham, United Kingdom).

### Construction of pLuxMC1, pLuxMC2 and pLuxMC3

The vector pPL2*lux *[2] containing a unique SwaI restriction site that overlaps the start codon of *luxA*, allowing translational fusions between bifidobacterial expression signals to the *lux*ABCDE operon, was digested with SalI and PstI to excise the 5.6 kb modified *lux*ABCDE operon. This was ligated into similarly digested pUC19 and cloned into TOP10. This newly created plasmid was named pUC19-*lux*. In order to create a control vector which does not contain a promoter in front of *lux*ABCDE, the operon was excised from pUC19-lux as a PstI/SmaI fragment, ligated into PstI and EcoRV-digested pBC1.2 [14] to create pLuxMC1, which was generated using TOP10 as the cloning host, and which was then introduced into *B. breve *UCC2003 by electroporation.

The putative promoter region of *repC *from the *B. catenulatum *plasmid pBC1 [1] was PCR amplified and digested with SalI. The resulting 0.5-kb PCR product (P_*rep*_) encompassed the region immediately upstream of the replication gene. Notably, the start codon of the *repC *was included at the ultimate 3' end of the reverse primer. This fragment was ligated into pUC19-*lux *digested with SwaI/SalI and cloned into TOP10. Isolates producing light (as monitored by the IVIS100, Xenogen) were subjected to restriction and PCR analysis. The plasmid content of one clone was subsequently sequenced and shown to represent the expected fusion. This plasmid was digested with PstI/SmaI, ligated into pBC1.2 [14] which had been digested with PstI/EcoRV, creating pLuxMC2, and cloned into TOP10. Plasmid DNA from a clone with the correct restriction profile was concentrated (1 μg) and electroporated in *B. breve *UC2003. A single chloramphenicol-resistant transformant was selected based on its verified genotype and used for subsequent bioluminescence activity determinations.

The promoter P_*CP*25 _is a highly active, constitutive lactococcal consensus promoter [47] which was synthesized by 'gene tiling' [48]. Riedel *et al *[3] exploited P_*CP*25 _to create P_*help *_(**h**ighly **e**xpressed ***L****isteria ***p**romoter) by introducing the 5' UTR (untranslated region) of the *L. monocytogenes *EGDe *hly*A gene into P_*CP*25 _using the 'gene tiling' approach. The P_*help *_promoter was cloned into pPL2*lux *[2] as an exact translational fusion to *lux*ABCDE creating pPL2*lux*Phelp. This modified *lux*ABCDE operon, containing the constitutive P_*help *_promoter, was excised from pPL2*lux*Phelp as an XhoI/PstI fragment and ligated into pBC1.2 digested with PstI/SalI, creating pLuxMC3, and cloned into TOP10. In the same manner as for pLuxMC2, plasmid DNA from a pLuxMC3 clone with the correct restriction profile was electroporated in *B. breve *UC2003 and assessed for subsequent bioluminescence activity assays.

### Plasmid stability studies

*B. breve *UCC2003 containing pLuxMC1, pLuxMC2 or pLuxMC3 were first cultured in MRS broth containing 4 μg ml^-1 ^chloramphenicol (Cm). Cells were then subcultured in fresh MRS broth without antibiotic selection for a total of 100 generations. Vector segregation stability was monitored by plating for isolated colonies every 20 generations and spot inoculating 100 colonies onto RCA agar plates with and without 4 μg ml^-1 ^Cm and incubating at 37°C for 24 h. The percentage loss of the test plasmid in the population was then calculated.

### Oral inoculation of mice

Inocula were prepared by growing *B. breve *UCC2003 containing either pLuxMC2, pLuxMC3 or the control vector pLuxMC1 anaerobically overnight at 37°C in 100 ml of MRS broth containing 4 μg ml^-1 ^Cm. Strains were tested in 6–8 week old female BALB/c mice. Animals were kept in a conventional animal colony and all experiments were approved by the animal ethics committee of University College Cork. Cultures were harvested by centrifugation (7,000 × *g *for 5 min), washed with PBS supplemented with 0.05% cysteine HCl (Sigma), and resuspended in a one-tenth volume of PBS. Colonization of bifidobacteria was established by three consecutive daily administrations whereby each animal received 20 μl of ~10^9 ^cells using a micropipette tip placed immediately behind the incisors [49]. The viable count of each inoculum was determined by retrospective plating on RCA agar containing 4 μg ml^-1 ^Cm.

### Comparison of pLuxMC2 and pLuxMC3 *in vivo*

To assess the functionality of the pLuxMC plasmids *in vivo*, three groups, each containing three eight-week old female BALB/c mice, were orally inoculated with *B. breve *UCC2003 containing either pLuxMC1 (negative control), pLuxMC2 or pLuxMC3. Three days post-inoculation the animals were anesthetised with isofluorane and whole-body image analysis was performed in the Xenogen IVIS 100 system for 5 minutes at high sensitivity. Stool samples were recovered aseptically on each of the feeding days (day 1 to 3) and subsequently on days 5, 8, 10 and 12, and examined for bioluminescence using a Xenogen IVIS 100 system, weighed and resuspended in PBS at 0.1 g ml^-1^. In order to estimate the number of bifidobacteria harbouring a pLuxMC derivative per gram of faeces, individual faecal samples were serially diluted and cultured on selective agar (RCA containing Cm 4 μg ml^-1^). On day 12 the animals were sacrificed by cervical dislocation, their individual gastrointestinal (GI) tracts were removed and these were examined for bioluminescence. Following imaging, the small intestine, caecum and large intestines were individually homogenized in sterile PBS supplemented with 0.05% cysteine-HCl and serial dilutions were plated in duplicate onto RCA agar containing 4 μg ml^-1 ^Cm. The resulting colonies were used to calculate the number of bacterial cells per tissue sample.

### Persistence of *B. breve *UCC2003::pLuxMC2 in the murine model

Following the comparative study detailed above, *B. *breve UCC2003 pLuxMC2 was selected to investigate the persistence of the strain in an animal model over a 33-day period. Two groups, each containing five eight-week old female BALB/c mice, were orally inoculated on day 1 to 3 as described in the previous section with either *B. breve *UCC2003 harbouring pLuxMC1 or *B. breve *UCC2003 containing pLuxMC2. Stool samples were recovered aseptically three times a week for five weeks post-inoculation, examined for bioluminescence using the Xenogen IVIS 100 system, weighed and resuspended in PBS at 0.1 g ml^-1^. In order to estimate the number of *B. breve *UCC2003 cells per gram of faeces, individual faecal samples were serially diluted and cultured on selective agar (RCA Cm 4 μg ml^-1^).

At selected time points, initially day 5 (two days post-feeding), and subsequently every 7 days (day 12, 19, 26 and 33), one animal per group was sacrificed by cervical dislocation, followed by the removal of the gastrointestinal (GI) tract, which was then examined for bioluminescence. The small intestine, caecum and large intestines were individually homogenized in sterile PBS supplemented with 0.05% cysteine-HCl and serial dilutions were plated in duplicate onto RCA agar containing Cm to calculate the number of *B. breve *UCC2003 cells per tissue sample. Following enumeration of *B. breve *UCC2003 cells in tissue or faecal samples, 100 random colonies were tested for the presence of the plasmid by colony PCR using primers IM111 and IM112 (Table 1). The contribution of the native flora to the total bifidobacterial population was also estimated, by plating dilutions from tissue samples onto RCA containing mupirocin (Oxoid). After incubation, random isolates were spot inoculated onto RCA containing either Cm 4 μg ml^-1 ^or mupirocin; colonies failing to replicate on RCA Cm 4 μg ml^-1 ^were predicted not to be *B. breve *UCC2003 though typical bifidobacterial morphology was observed following microscopic analysis. Species identification of these host-specific strains was achieved using primers targeting the spacer region [32].

## Authors' contributions

MC conceived of the study, carried out the molecular genetic studies and drafted the manuscript. RDS carried out the animal studies, assisted with statistical analysis and helped to draft the manuscript. CH helped to draft the manuscript. GFF participated in the design of the study and helped to draft the manuscript. DvS participated in the design and coordination of the study and helped to draft the manuscript. All authors read and approved the final manuscript.

## References

[B1] Alvarez-Martin P, Florez AB, Mayo B (2007). Screening for plasmids among human bifidobacteria species: Sequencing and analysis of pBC1 from *Bifidobacterium catenulatum *L48. Plasmid.

[B2] Bron PA, Monk IR, Corr SC, Hill C, Gahan CG (2006). Novel luciferase reporter system for in vitro and organ-specific monitoring of differential gene expression in *Listeria monocytogenes*. Appl Environ Microbiol.

[B3] Riedel CU, Monk IR, Casey PG, Morrissey D, O'Sullivan GC, Tangney M, Hill C, Gahan CG (2007). Improved luciferase tagging system for *Listeria monocytogenes *allows real-time monitoring in vivo and in vitro. Appl Environ Microbiol.

[B4] Diaz RL, Hoang LH, Wang J, Vela JL, Jenkins S, Aranda R, Martin MG (2004). Maternal adaptive immunity influences the intestinal microflora of suckling mice. J Nutr.

[B5] Winkler P, Ghadimi D, Schrezenmeir J, Kraehenbuhl JP (2007). Molecular and cellular basis of microflora-host interactions. J Nutr.

[B6] Duffy LC, Leavens A, Griffiths E, Dryja D (1999). Perspectives on bifidobacteria as biotherapeutic agents in gastrointestinal disease. Dig Dis Sci.

[B7] Liévin V, Peiffer I, Hudault S, Rochat F, Brassart D, Neeser JR, Servin AL (2000). Bifidobacterium strains from resistant infant human gastrointestinal microflora exert antimicrobial activity. Gut.

[B8] Sheehan VM, Sleator RD, Hill C, Fitzgerald GF (2007). Improving gastric transit, gastrointestinal persistence and therapeutic efficacy of the probiotic strain *Bifidobacterium breve *UCC2003. Micro.

[B9] Menard O, Butel MJ, Gaboriau-Routhiau V, Waligora-Dupriet AJ (2008). Gnotobiotic mouse immune response induced by *Bifidobacterium *sp. strains isolated from infants. Appl Environ Microbiol.

[B10] Ventura M, van Sinderen D, Fitzgerald GF, Zink R (2004). Insights into the taxonomy, genetics and physiology of bifidobacteria. Antonie Leeuwenhoek.

[B11] Ventura M, O' Connell-Motherway M, Leahy S, Moreno-Munoz JA, Fitzgerald GF, van Sinderen D (2007). From bacterial genome to functionality; case bifidobacteria. Int J Food Microbiol.

[B12] Cronin M, Knobel M, O'Connell-Motherway M, Fitzgerald GF, van Sinderen D (2007). Molecular dissection of a bifidobacterial replicon. Appl Environ Microbiol.

[B13] Sangrador-Vegas A, Stanton C, van Sinderen D, Fitzgerald GF, Ross RP (2007). Characterisation of plasmid pASV479 from *Bifidobacterium pseudolongum *subsp. *globosum *and its use for expression vector construction. Plasmid.

[B14] Alvarez-Martin P, O'Connell-Motherway M, van Sinderen D, Mayo B (2007). Functional analysis of the pBC1 replicon from *Bifidobacterium catenulatum *L48. Appl Microbiol Biotechnol.

[B15] Bron P, Hoffer ASM, Van Swam II, de Vos W, Kleerebezem M (2004). Selection and characterization of conditionally active promoters in *Lactobacillus plantarum*, using alanine racemase as a promoter probe. Appl Environ Microbiol.

[B16] Oozeer R, Furet JP, Goupil-Feuillerat N, Anba J, Mengaud J, Corthier G (2005). Differential activities of four *Lactobacillus casei *promoters during bacterial transit through the gastrointestinal tracts of human-microbiota-associated mice. Appl Environ Microbiol.

[B17] Platteeuw C, Simons G, de Vos WM (1994). Use of the *Escherichia coli *β-glucuronidase (gusA) gene as a reporter gene for analyzing promoters in lactic acid bacteria. Appl Environ Microbiol.

[B18] Klijn A, Moine D, Delley M, Mercenier A, Arigoni F, Pridmore RD (2006). Construction of a Reporter Vector for the Analysis of *Bifidobacterium longum *Promoters. Appl Environ Microbiol.

[B19] Mazé A, O'Connell-Motherway M, Fitzgerald GF, Deutscher J, van Sinderen D (2007). Identification and characterization of a fructose phosphotransferase system in *Bifidobacterium breve *UCC2003. Appl Environ Microbiol.

[B20] Ventura M, Zhang Z, Cronin M, Canchaya C, Kenny JG, Fitzgerald GF, van Sinderen D (2005). The ClgR protein regulates transcription of the clpP operon in *Bifidobacterium breve *UCC2003. J Bacteriol.

[B21] Greer LF, Szalay AA (2002). Imaging of light emission from the expression of luciferases in living cells and organisms: a review. Luminescence.

[B22] Francis K, Joh PD, Bellinger-Kawahara C, Hawkinson MJ, Purchio TF, Contag PR (2000). Monitoring bioluminescent *Staphylococcus aureus *infections in living mice using a novel luxABCDE construct. Infect Immun.

[B23] Hutchens M, Luker GD (2007). Applications of bioluminescence imaging to the study of infections diseases. Cell Microbiol.

[B24] Qazi SN, Counil E, Morrissey J, Rees CE, Cockayne A, Winzer K, Chan WC, Williams P, Hill PJ (2001). agr expression precedes escape of internalized *Staphylococcus aureus *from the host endosome. Infect Immun.

[B25] Hardy J, Francis KP, DeBoer M, Chu P, Gibbs K, Contag CH (2004). Extracellular replication of *Listeria monocytogenes *in the murine gall bladder. Science.

[B26] Francis KP, Yu J, Bellinger-Kawahara C, Joh D, Hawkinson MJ, Xiao Purchio GF, Caparon MG, Lipsitch M, Contag PR (2001). Visualizing pneumococcal infections in the lungs of live mice using bioluminescent *Streptococcus pneumoniae *transformed with a novel gram-positive lux transposon. Infect Immun.

[B27] Guglielmetti S, Ciranna A, Mora D, Parini C, Karp M (2008). Construction, characterisation and exemplificative application of bioluminescent *Bifidobacterium longum *biovar *longum*. Int J Food Microbiol.

[B28] Rice BW, Cable MD, Nelson MB (2001). *In vivo *imaging of light-emitting probes. J Biomed Opt.

[B29] Wiles S, Clare S, Harker J, Huett A, Young D, Dougan G, Frankel G (2004). Organ specificity, colonization and clearance dynamics in vivo following oral challenges with the murine pathogen *Citrobacter rodentium*. Cell Microbiol.

[B30] Simpson PJ, Stanton C, Fitzgerald GF, Ross PR (2003). Genomic Diversity and Relatedness of Bifidobacteria Isolated from a Porcine Cecum. J Bacteriol.

[B31] Riedel CU, Casey P, Mulcahey H, O'Gara F, Gahan CGM, Hill C (2007). Construction of p16S*lux*, a novel vector for improved bioluminescent labeling of gram-negative bacteria. Appl Environ Microbiol.

[B32] Haarman M, Knol J (2005). Quantitative real-time PCR assays to identify and quantify faecal *Bifidobacterium *species in infants receiving a probiotic infant formula. Appl Environ Microbiol.

[B33] Ryan S, Fitzgerald GF, van Sinderen D (2004). Screening for and identification of starch-, amylopectin-, and pullulan-degrading activities in 8 bifidobacterial strains. Appl Environ Microbiol.

[B34] Boyandin AN, Popova LY (2003). Expression of lux-genes as an indicator of metabolic activity of cells in model ecosystem studies. Adv Space Res.

[B35] Szittner R, Meighen E (1990). Nucleotide sequence, expression, and properties of luciferase coded by lux genes from a terrestrial bacterium. J Biol Chem.

[B36] Rada V, Sirotek K, Petr J (1999). Evaluation of selective media for bifidobacteria in poultry and rabbit caecal samples. Zentralbl Veterinarmed B.

[B37] Marteau P, Pochart P, Dore J, Bera-Maillet C, Bernalier A, Corthier G (2001). Comparative study of bacterial groups within the Human cecal and fecal microbiota. Appl Environ Microbiol.

[B38] Dean-Nystrom EA, Bosworth BT, Moon HW (1999). Pathogenesis of *Escherichia coli *O157:H7 in weaned calves. Adv Exp Med Biol.

[B39] Siebers A, Finlay BB (1996). M cells and the pathogenesis of mucosal and systemic infections. Trends Microbiol.

[B40] Baumler AJ, Tsolis RM, Heffron F (1996). The lpf fimbrial operon mediates adhesion of *Salmonella typhimurium *to murine Peyer's patches. Proc Natl Acad Sci USA.

[B41] Mundy R, Pickard D, Wilson RK, Simmons CP, Dougan G, Frankel G (2003). Identification of a novel type IV pilus gene cluster required for gastrointestinal colonization of *Citrobacter rodentium*. Mol Microbiol.

[B42] Kingsley RA, Santos RL, Keestra AM, Adams LG, Baumler AJ (2002). *Salmonella enterica *serotype *Typhimurium *ShdA is an outer membrane fibronectin-binding protein that is expressed in the intestine. Mol Microbiol.

[B43] Kleessen B, Blaut M (2005). Modulation of gut mucosal biofilms. Br J Nutr.

[B44] Miller JH (1972). Experiments in molecular genetics.

[B45] MacConaill LE, Fitzgerald GF, van Sinderen D (2003). Investigation of protein export in *Bifidobacterium breve *UCC2003. Appl Environ Microbiol.

[B46] Sambrook J, Fritsch EF, Maniatis T (1989). Molecular cloning: a laboratory manual.

[B47] Jensen PR, Hammer K (1998). The sequence of spacers between the consensus sequences modulates the strength of prokaryotic promoters. Appl Environ Microbiol.

[B48] Kodumal SJ, Patel KG, Reid R, Menzella HG, Welch M, Santi DV (2004). Total synthesis of long DNA sequences: synthesis of a contiguous 32-kb polyketide synthase gene cluster. Proc Natl Acad Sci USA.

[B49] Sleator RD, Gahan G, Hill C (2001). Identification and disruption of the proBA locus in *Listeria monocytogenes*: role of proline biosynthesis in salt tolerance and murine infection. Appl Environ Microbiol.

